# Genetic Determinants of Biomass in C_4_ Crops: Molecular and Agronomic Approaches to Increase Biomass for Biofuels

**DOI:** 10.3389/fpls.2022.839588

**Published:** 2022-06-23

**Authors:** Noor-ul- Ain, Fasih Ullah Haider, Mahpara Fatima, Habiba, Yongmei Zhou, Ray Ming

**Affiliations:** ^1^Fujian Provincial Key Laboratory of Haixia Applied Plant Systems, FAFU and UIUC-SIB Joint Center for Genomics and Biotechnology, College of Crop Sciences, Fujian Agriculture and Forestry University, Fuzhou, China; ^2^College of Resources and Environmental Sciences, Gansu Agricultural University, Lanzhou, China; ^3^Fujian Provincial Key Laboratory of Plant Functional Biology, College of Life Science, Fujian and Agriculture and Forestry University, Fujian, China; ^4^Department of Plant Biology, The University of Illinois at Champaign-Urbana, Champaign, IL, United States

**Keywords:** biomass accumulation, C_4_ crops, hormone dynamics, cell wall growth, circadian rhythms, fossil fuels

## Abstract

Bio-based fuels have become popular being efficient, cost-effective, and eco-friendly alternatives to fossil fuels. Among plant sources exploited as feedstocks, C_4_ grasses, such as sugarcane, maize, sorghum, and miscanthus, are highly resourceful in converting solar energy into chemical energy. For a sustainable and reliable supply of feedstocks for biofuels, we expect dedicated bioenergy crops to produce high biomass using minimum input resources. In recent years, molecular and genetic advancements identified various factors regulating growth, biomass accumulation, and assimilate partitioning. Here, we reviewed important genes involved in cell cycle regulation, hormone dynamics, and cell wall biosynthesis. A number of important transcription factors and miRNAs aid in activation of important genes responsible for cell wall growth and re-construction. Also, environmental components interacting with genetic controls modulate plant biomass by modifying gene expression in multiple interacting pathways. Finally, we discussed recent progress using hybridization and genome editing techniques to improve biomass yield in C_4_ grasses. This review summarizes genes and environmental factors contributing biomass yield in C_4_ biofuel crops which can help to discover and design bioenergy crops adapting to changing climate conditions.

## Introduction

The world population is expected to reach 9 billion by 2050, which was only ∼1.9 billion in the twentieth century (Mullet, 2017). Rapid population expansion and rise in energy demands have heightened research interests to build more sustainable, cost-effective, and eco-friendly energy sources. The gradual effects of using rapidly depleting finite fossil fuels turned out in global warming (greenhouse effect) and subsequent climate change that is ongoing. Owing to intensive industrialization and urbanization, the level of greenhouse gases (GHGs) has increased by 50 times in the atmosphere, which is among the primary drivers of climate change (Subramaniam et al., 2020). International Energy Agency predicted that from 2020 to 2025, the mean annual near-surface temperature would increase by 1°C with a range of 0.9–1.8°C in comparison with pre-industrial time-temperature level (time spanning from 1850 to 1900) along with the onset of frequent tropical cyclones (IEA, 2018). To minimize the disastrous effects of climate change and fulfill energy demands, International Energy Agency (IEA) has devised to exploit renewable energy sources. Major renewable energy resources, such as wind, solar, plant biomass, ocean, and hydropower being sustainable sources, can also aid in reducing the use of fossil fuels and consequently GHGs. In the year 2019, a 12.5% increase in biofuel production was observed from 142.6 to 160.9 million liters, whereas in 2020 due to the global pandemic situation, compromised prices of fossil crude oil have made transport biofuels less competitive. However, future predictions suggest that the average global output of bioethanol will further rise to 182 billion liters which was 160 million liters in 2019, whereas the United States and ASEAN region will contribute for biodiesel and Hydro-treated vegetable oil (HVO) (Lauf et al., 2021).

Biofuels are the fuels derived from biological substances (e.g., agricultural wastes, animal matter, algae, forest vegetation, and energy crops) (Koçar and Civaş, 2013). In this review, we will primarily focus on the high biomass yielding energy crops which are commercially used for energy generation. Presently, regarding biofuel crops, there is a concern of conflict about their use for food, feed, and energy generation. However, several plant species are efficient in accumulating high biomass with minimal inputs. Among them, ryegrass (*Lolium perenne*), bamboo (*Bambusa vulgaris*), poplar (*Populus deltoides*), and willow (*Salix*) are from C_3_ category of photosynthesis while giant miscanthus (*Miscanthus giganteus*), sweet sorghum (*Sorghum bicolor*), pearl millet (*Pennisetum glaucum*), napier grass (*Pennisetum purpureum*), maize (*Zea mays*), sugarcane (*Saccharum*), and switchgrass (*Panicum virgatum*) are ideal C_4_ energy crops (Byrt et al., 2011). C_4_ grasses take more advantage of biomass accumulation, owing to higher energy-conserving photosynthetic machinery, stress tolerance, water, and nitrogen use efficiency (Somerville et al., 2010). C_4_ photosynthesis system possesses specialized biochemistry and anatomical modifications that protect the oxygenation of RUBISCO. Whereas, the PEP enzyme, instead of RUBISCO serves as the first substrate of CO_2_ in mesophyll cells that reduces the energy losses caused by photorespiration (van der Weijde et al., 2013). This structural collaboration of mesophyll (M) and bundle sheath (BS) enables C_4_ plants to harvest more solar energy with improved water use efficiency (WUE) and nitrogen use efficiency (NUE).

In a couple of decades, the increasing trend of biofuel use in developed countries motivated many researchers to focus their interests on biofuel crops and their bio-products. Among C_4_ crops, Miscanthus and switchgrass have been extensively studied for this purpose in the United States and Europe. Sugarcane is contributing a major share of biofuels in Brazil. Sorghum is also a promising competitor as a bio-energy crop owing to drought tolerance and genetic diversity in sweet and grain sorghum (Byrt et al., 2011). Advancement in recent technologies of saccharification and lignocellulose digestion, cultivation of sugarcane is widely practiced as a biofuel crop (Carvalho-Netto et al., 2014). Scientists are more concerned with introducing specialized hybrids or transgene as energy crops to meet the objectives of sustainable energy by biofuels. Plant biomass depends on genetic, physiological, edaphic and environmental factors. Few publications encompassed basis of biomass accumulation at genetic level but a comprehensive study of genetic, physiological and environmental factors constituting to biomass, was lacking (Endo et al., 2009; Byrt et al., 2011; Lima et al., 2017). Therefore, besides biological and genetic basis we addressed environmental modulations-based plant biomass accumulation. Considering the general overview of the need for bioenergy and the importance of biomass crops with special concern for C_4_ crops, this review aims at identifying the genes involved in different growth-related processes and how growth patterns are modified in changed climatic conditions. Furthermore, possible tools and strategies are discussed that are effective in opting for increasing biomass in C_4_ crops.

## Genetic Basis for Biomass

In plants, several genes belonging to different functional and structural categories are involved in vegetative development. Owning to advancement in molecular and genetic studies, many genes and transcription factors have been identified in contributing growth from juvenile to vegetative stage as previously covered in the reviews (Demura and Ye, 2010; Lima et al., 2017; Kandel et al., 2018). Following sections supplicated the existing literature about genetic aspects of plant growth regulation.

### Growth and Developmental Regulation

#### Cell Cycle Machinery

Plant organ size control is a central component of biomass productivity. In many animals and plants, overall organ growth rate and size are associated with cell number that is controlled by strict action of cell division together with cell expansion (Vernoux et al., 2000). As in other eukaryotes, the cell cycle in plants consists of DNA-replication (S-phase), and mitosis (M-phase), which are separated by postmitotic (G1) and pre-mitotic interphase (G2) gap phases, respectively (Scofield et al., 2014). Cell cycle machinery is strongly modulated at different points to verify the fidelity of chromosome duplication and cell division. Highly conserved control mechanisms, the checkpoints G1/S and G2/M transitions confirm that either cell cycle process has been precisely accomplished at each phase before entering the next phase or not (Barnum and O’Connell, 2014). Different core cell cycle protein groups including CYCLINS (CYCs) complexed with CYCLIN-DEPENDENT KINASES (CDKs), the E2F/DIMERISATION PROTEIN (DP) transcriptional regulatory proteins, KIP-RELATED PROTEIN/INTERACTOR OF CDKs (KRP/ICK), RETINOBLASTOMA-RELATED (RBR), SIAMESE/SIAMESE-RELATED (SIM/SMR), proteins and the multi-subunit E3 ubiquitin ligase ANAPHASE-PROMOTING COMPLEX/CYCLOSOME (APC/C) control the progression of events involved different phases (Inz, 2006; Inagaki and Umeda, 2011). Genetic modulation of these proteins to enhance biomass has been reported in model plant species (*Arabidopsis*, tobacco, rice), with few C_4_ plants (sorghum and maize). Elevated growth rate in tobacco has been resulted from overexpressing the cyclin D-type (*CycD2*) gene from *Arabidopsis*. These plants were found to have normal cell and meristem size but taller stem overall, showing increased growth rate from seedling to maturity (Boucheron et al., 2005). Defective shoot and root formation, as well as a reduction in endoreduplication, were noticed in tobacco ectopically expressing *CycA3; 2* (Yu et al., 2003). Transcriptome analysis identified elevated levels of cell cycle (i.e., cyclins) genes in bioenergy sorghum immature internodes which shows that their initial increase in size is due to cell-division coupled growth (Kebrom et al., 2017). Further, quadruple (*ick1/krp1*, *ick2/krp2*, *ick6/krp3*, *ick7/krp4*) and quintuple (*ick1/krp1*, *ick2/krp2*, *ick6/krp3*, *ick7/krp4*, *ick5/krp7*) mutants of CDK negative regulator *ICK/KRP* genes reported to have stimulated CDK activity and cell proliferation that resulted in increased fresh and dry weights; larger cotyledons; leaves; petals and seeds (Cheng et al., 2013). Overexpression of novel *Arabidopsis* ABAP1 protein decreased cell proliferation by limiting mitotic DNA replication in negative feedback loop during leaf development, by repressing transcription of pre-replication complex (pre-RC) genes (Masuda et al., 2008).

Another essential component of cell cycle machinery is the anaphase-promoting complex (APC), a multi-subunit E3 ligase that modulates cyclins (Cyc) and CDKs activity in checkpoints to ensure the maintenance of cell division rate (Bodrug et al., 2021). APC/C-subunits remain conserved throughout evolution, however, gene duplication of different subunits has been observed in some plants (de Lima et al., 2010). Overexpression of *Arabidopsis CDC27a/APC3a* in tobacco was associated with apical meristem restructuration, altered cell-cycle marker expression, and accelerated plant growth up to 30% at the flowering time leading to increased biomass production (Rojas et al., 2009). While *APC10* overexpression in *Arabidopsis* causes CYCB1;1 protein degradation, thereby accelerating the transition through mitosis (Eloy et al., 2011). Transgenic tobacco plants overexpressing the *APC10* gene are taller with larger leaves, produce more seed capsules, and have an augmented biomass accumulation. Furthermore, a cross between *APC3a-* and *APC10-* overexpressing tobacco T1 plants have enhanced growth phenotype compared to the overexpression of single *APC/C* subunits (de Freitas Lima et al., 2013). Down-regulation of rice *OsCCS52A, an* APC/C subunit resulted in reduced plant height and smaller seeds with an endosperm defect in endoreduplication (Su’udi et al., 2012). Semi-dwarfism and reduced leaf size are also observed in *CCS52A* ortholog rice *tillering and dwarf 1* (*tad1*) mutant (Xu et al., 2012). SAMBA negatively modulates cell proliferation through APC/C interaction. In maize, *samba-1* and *samba-3* mutants showed developmental defects, involving short plant height, reduced leaf size due to an altered cell expansion and cell division rate (Gong et al., 2021). In addition, several DRP family members like DRP1A, DRP1E, DRP2A, DRP2B, and DRP5B are regarded as SAMBA interactors. All of these proteins, except DRP5B, are localized to the cell plate and mutations in *DRP1E* and *DRP1A* resulted in defective cell plate assembly and cytokinesis, as well as defects in cell expansion (Hong et al., 2003; Kang et al., 2003; Fujimoto et al., 2008).

#### Long Vegetative Duration

The plant life cycle is divided into vegetative, transition, and reproductive developmental phases. The vegetative phase is associated with meristems producing stems and leaves. The transition phase is related to an elevation of the apical meristem, while the reproductive phase centers on meristems capable of producing flowers or reproductive organs. The vegetative phase starts at germination and continues through tillering, the meristems actively produce a stem, buds, internodes, and leaves. The long vegetative phase establishes continuous leaf development needed to capture sunlight for photosynthesis that supplies nutrients for expansion of roots for anchoring, storage, and uptake of minerals for increased biomass production. Some high-yielding C_4_ crops are *Miscanthus x giganteus*, *Sorghum bicolor*, *Pennisetum*, and sugarcane (*Saccharum* spp.) genotypes, characterized by enriched canopies, taller stems, and longer growing seasons (Somerville et al., 2010; Mullet et al., 2014). The biomass yield of *Miscanthus × giganteus* in some mid-west United States locations reached 44–61 Mg/ha at peak biomass accumulation (Heaton et al., 2008). *Pennisetum purpureum* and *Pennisetum typhoides* reached their record yields of ∼88 and 80 Mg/ha, respectively during longer growing seasons (Somerville et al., 2010). Similarly, high-biomass first-generation sorghum hybrids accumulated ∼40–50 dry Mg/ha during ∼180 days growing season when grown in the south-central United States (Olson et al., 2012). Biomass varies among types of C_4_ crops for various reasons like stem sink strength, shoot/root partitioning, and season length. For example, *Miscanthus × giganteus* produced higher biomass than switchgrass and maize when these were grown in similar regions in the United States due to differences in shoot/root biomass partitioning (Anderson et al., 2011). High-biomass sorghum hybrids with long growing seasons produce twice shoot biomass (∼40–50 Mg/ha) when compared to grain sorghum hybrids in optimum growth conditions and ∼30% more biomass in rain-fed conditions when both hybrids are grown in similar regions in the south-central United States (Olson et al., 2012; Truong et al., 2017). The increased biomass yield of high-biomass sorghum hybrids was because of delayed flowering initiation resulting in prolonged vegetative growth duration that increased total light energy capture, improved radiation interception and use efficiency, and elevated biomass partitioning.

Further, delayed flowering in long days concomitant with an extensive period of vegetative growth resulted in increased biomass yield as observed in several C_4_ grasses. Photoperiod regulated flowering in sorghum is extensively studied by modulating flowering regulators florigen related genes (*CN8, CN12, CN15*), upstream activators (*CONSTANS (CO)* and *EARLY HEADING DATE 1* (*EHD1*)), and repressors *(PRR37* and *GHD7)* of these genes that are regulated by photoperiod and output from the circadian clock, once sorghum exits in juvenile phase (Murphy et al., 2011, 2014; Dong et al., 2012). Prolonged vegetative meristem activity with increased biomass yield was observed in several plants by overexpressing flowering-time genes (Demura and Ye, 2010). Indeed, the activation of the flowering promoting factor-like1, flowering locus T1, C-like MADS-box protein, early flowering 3 as well as embryo flowering 1-like protein in tomato IL2-6 cultivar, supported late-maturing performance (Caruso et al., 2016). Overexpression of gibberellin 20-oxidase-1 and ARGOS also resulted in an extended growing period by delaying the flowering time to give rise to larger organ size and taller plants (Hu et al., 2003; Voorend et al., 2016). Regulation switch from vegetative to reproductive phase can be controlled by manipulating genes from several developmental pathways, for example, gibberellin, circadian, and flowering-related genes.

#### Hormones Dynamics and Primary Growth

Plant hormones are a diverse group of chemical substances controlling growth and development-related events in plants by regulating meristematic cell division and cell elongation. These chemical signals modulate microtubule and cell plate formation, cell wall constituent deposition, and remodeling which are key factors of growth and thus biomass accumulation. During the green revolution, scientists exploited the traditional plant breeding approaches for the selection of short stature, higher grain yield producing cultivars, which were low in levels of endogenous hormones like gibberellin (Sánchez-Rodríguez et al., 2010), auxin (Vanneste and Friml, 2009), and brassinolide (Müssig, 2005) that are important as growth regulators and performing growth regulatory functions from cellular to developmental levels (Table 1).

**TABLE 1:** List of C_4_ crops genes as candidates for exploiting biomass-related traits.

Crop species	Gene/Enzyme manipulated	Description	Comments	References
**Cell cycle machinery**				
Tobacco	*CycA3;3*	A-type cyclins	Antisense expression led the formation of defective embryo and impaired callus formation	Yu et al., 2003
Tobacco	*Arath-CYCD2 or Arath-CYCD3*	D-type cyclins (G_1_-specific cyclins)	OE transgenics exhibited increased cell number but not cell size with higher leaf initiation rates	Boucheron et al., 2005
Maize	*Samba1* and *samba 3*	SAMBA	CRISPR/Cas9 mutant lines accelerated cell cycle, erect and shortened foliage upper top leaf length, ligule formation and internode elongation	Gong et al., 2021

**Hormone related genes**				
*Arabidopsis*	*GA20ox*	GA20-oxidase	OE produced 25% taller plants, accelerated shoot growth and early flowering	Coles et al., 1999
Switchgrass	*GA20ox*	GA20-oxidase	OE lines showed 2 folds biomass increase due to more tillers, leaf size and elevated bioactive GAs	Do et al., 2016
Sorghum	*GA20ox1*	GA20-oxidase	Higher expression in bioenergy sorghum culms. Moreover, sweet sorghum had higher GA levels and biomass.	Wang et al., 2020
Sugarcane	*GAI*	DELLA repressors	OE lines showing the stunted culm growth and development and modulation of shoot-to-root ratio in sugarcane	Garcia Tavares et al., 2018
Maize	*AUX1*	(AUX/LAX) Auxin influx facilitators	Mutant showed inflorescence development and root gravitropism	Huang et al., 2017
Green foxtail	*AUX1*	(AUX/LAX)	Mutants led to defective inflorescence, reduced plant height, increased panicle length and sparse panicle phenotype	Huang et al., 2017
Maize	*Aux/IAA*	RUM1 (ROOTLESS WITH UNDETECTABLE MERISTEMS 1)	Mutant *rum1* showed defective xylem organization and more lignin deposition in root cells	Zhang et al., 2015
Maize	*ARF5*	(MONOPTEROS)	Mutant showed root altered patterning of vascular cells differentiation, thick cell walls with higher lignin contents	Zhang et al., 2014
Maize	*D11*	Biosynthesis of BL	Higher expression in young ears and seeds, Improve seed quantity and quality.	Sun et al., 2021
Maize	*BRI1*	BRASSINOSTEROID INSENSITIVE 1	Mutant displayed overall dwarf stature, shortened internodes, folded dark green leaves, decreased auricle formation and feminization of female flowers	Kir et al., 2015

**Cell wall biosynthesis related genes**				
*Arabidopsis*	*xxt1 xxt2*	Xyloglucan transferase	Double mutant showed aberrant root hairs and modified mechanical properties	Cavalier et al., 2008
*Arabidopsis*	*TED6 and TED7*	Tracheary Element (TE) Differentiation-Related 6 and 7	RNAi showed delay in TE differentiation, abnormality in SCW and cellulose synthesis	Endo et al., 2009
*Arabidopsis*	*LAC4 & LAC17*	Laccases	T-DNA insertion showed LAC17 effected the deposition of G lignin units in the interfascicular fiber. *lac4-2 lac17* double mutant resulted in 40% reduced lignin.	Berthet et al., 2011

**Plant species**	**Gene/Enzyme manipulated**	**Description**	**Comments**	**References**

Sugarcane	*LAC*	Laccases	Complementation in *Arabidopsis* lac17 (–19% lignin) mutant restored lignin content	Cesarino et al., 2013
Sorghum	*(bmr*) *bmr2*, *bmr6*, and *bmr12*	Brown midrib mutant	2 years-based field study of EMS *bmr* mutants displayed decreased levels of lignin	Sattler et al., 2014
Maize	(bm3) *COMT*	Brown-midrib-3, lacking caffeic acid O-methyltransferase (*COMT*)	Antisense (AS225), and bm3 maize plants resulted in disturbed cell wall assembly.	Guillaumie et al., 2008
Sorghum	*CslF6*	Cellulose synthase-like F6 (CslF6) Glucan biosynthesis	Chimeric cDNA construct modifies the fine structure of (1,3;1,4)-β-glucan polysaccharide chain	Dimitroff et al., 2016

**Transcription factors and MicroRNA**				
Switch grass	*ERF001*	SHINE “SHINE/WAX INDUCER” (SHN/WIN) TF (AP2/ERF) superfamily	Increased biomass, and efficient saccharification process	Wuddineh et al., 2015
Sugarcane	*SHN1*	SHINE “SHINE/WAX INDUCER” (SHN/WIN) TF e factor (AP2/ERF) superfamily	OE results modified cell walls and increase in biomass by (91–340%),	Martins et al., 2018
Maize	*MYB46/83*	MYB (myeloblastosis)	Synthesis and thickening of cell wall	Zhong et al., 2011
Switchgrass	*R2R3-MYB*	MYB (myeloblastosis)	OE lines showed an increased biomass up to ∼ 63% and reduced lignin content around 50%	Shen et al., 2012
Maize	*MYB31* and *MYB42*	MYB (myeloblastosis)	Redirected phenylpropanoid and lignin biosynthesis in *Arabidopsis*, reduced S/G ratio (S, syringl units; G, guaiacyl units)	Sonbol et al., 2009; Vélez-Bermúdez et al., 2015
Sorghum	*Myb60*	Myeloblastosis (*MYB*)	Overexpressed lines displayed enhanced lignification in leaf midribs and increased phenolics	Scully et al., 2016
Finger millet	*bHLH57*	*(BASIC* HELIX-LOOP-HELIX)	Over-expressing depicted resistance to salinity stress with enhanced photosynthetic efficiency and increased biomass	Babitha et al., 2015
Maize	*Dof 1*	Zinc finger protein	Increased NUE in transgenic sorghum and wheat. Activation of carbon skeleton metabolism, i.e., PEPC activity	Peña et al., 2017
Maize	miR156, AtSPL9, *MIR172*	*SQUAMOSA Promoter-Binding Protein-Like* (*SPL*) miRNA	Delays reproductive phase leading the prolonged vegetative stage	Lauter et al., 2005; Chuck et al., 2007
Switchgrass	GAUT4-KD, miRNA156-OE, MYB4-OE, COMT-KD and FPGS-KD).	Myeloblastosis (*MYB*) miRNA	Increased contents of carbohydrates by 12% and ethanol yields by 21%	Dumitrache et al., 2017

#### Gibberellin

In Gibberellin (GA) signaling pathways, manipulation of both positive and negative regulators employed positive effects on growth and biomass accumulation. One control point of biomass can be the substantial increase of GA rate-limiting enzyme GIBBERELLIN 20-OXIDASE (GA 20-OXIDASE) which is involved in the last steps of GA biosynthesis in the cytoplasm. One of the primitive functional evidence of *GA20ox* in *Arabidopsis* highlights the accelerated shoot growth, elongated hypocotyls, and onset of early flowering (Coles et al., 1999). In a potential biofuel crop, switchgrass, ectopic expression of *ZmGA20ox* resulted in increased growth and biomass-related traits (Do et al., 2016). Recently, nine genes of *GA20-ox1* were identified in sweet sorghum (bioenergy sorghum) and showed differential spatiotemporal patterns of expression while *SbGA20ox1* was predominantly related to increased stem biomass and assimilates partitioning (Wang et al., 2020). “Green revolution” gene *GA20-oxidase* is involved in the synthesis of principal biopolymer in the cell wall, i.e., cellulose in sorghum, whereas *dwarf1-1* cellulose deficient and male gametophyte-dysfunctional mutant showed ablation of GA and altered expression of three *CESA* genes generating cellulose deficient and dwarf phenotypes (Petti et al., 2015). Similarly, plants overexpressing *ZmGA20ox* displayed longer internodes and leaves, more tillers, and twofold increase in maize biomass (Voorend et al., 2016). Secondly, GA-INSENSITIVE DWARF1 (*GID1*) is the first receptor of bioactive GA in the signaling pathway, which shows the highest affinity for GA4 (bioactive form). Overexpressing the *GID1* gene shows a substantial increase in shoot elongation and growth in *Arabidopsis*, rice, and poplar (Sakamoto et al., 2004; Hirano et al., 2008; Mauriat and Moritz, 2009). The third main player in the gibberellin signaling pathway is the DELLA repressor gene which hinders the transcription of GA receptor, i.e., GID1. DELLA proteins act as a feedback regulatory control in the GA signaling pathway and are implicated in dwarf phenotype in maize with shifts in flowering time (Lawit et al., 2010). A recently conducted study on sugarcane affirmed the similar inhibitory roles of DELLA proteins. DELLA proteins interact with PIF4 and elements in the ethylene signaling pathway *ScEIN3/ScEIL1*, moreover, DELLA silenced lines showed changes in carbon allocation in storage and structural molecules and increased culm growth (Garcia Tavares et al., 2018).

#### Auxin

Auxin is a very important hormone involved in the growth process and cell wall architecture. Numerous mutants related to auxin synthesis, transport, and signaling showed overall dwarf phenotypes, defects in tropisms, and altered organ morphology (Vanneste and Friml, 2009). Auxin influx facilitator AUXIN1/LIKE-AUX1 (*AUX/LAX*) is involved in inflorescence development and root gravitropism. It is reported that mutations in homologs of AUX1 genes in maize (*ZmAUX1*) and *Setaria viridis* (*SvAUX1)* resulted in defective branch development of inflorescence, reduced plant height, increased panicle length, and sparse panicle phenotype (Huang et al., 2017). Aux/IAA homolog ROOTLESS WITH UNDETECTABLE MERISTEMS 1 (*RUM1*) in maize is involved in seminal and lateral roots formation. Transcriptome analysis of *rum1* showed down-regulated expression of like-auxin1 *(lax1)*, the plethora genes plethora 1 *(plt1)*, baby boom 1 *(bbm1)*, and heat shock complementing factor 1 *(hscf1*), and the auxin response factors *arf8 and arf37* (Zhang et al., 2015). In maize, *ARF5* (MONOPTEROS) is involved in vascular cells differentiation and *rum1* showed defective xylem organization and more lignin deposition in root cells (Zhang et al., 2014).

#### Brassinosteroid

**FIGURE 1:**
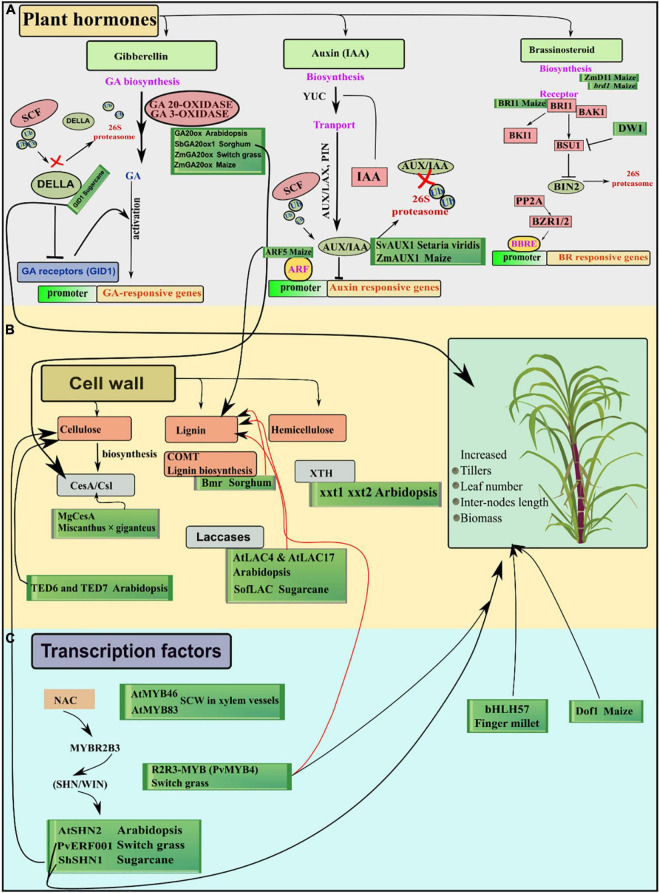
Overview of the genes involved in different pathways in important C_4_ biomass plants. **(A)** Highlights the hormone genes directly or indirectly related to growth and cell wall functioning. **(B)** Shows multiple genes involved in biosynthesis and remodeling of different cell wall-related components. **(C)** Transcription factors regulate many genes involved in different pathways of secondary cell wall synthesis leading to modified cell wall components improving saccharification efficiency. Black arrows show positive, red arrows show negative growth impacts and T lines show inhibitory influence on other genes. Whereas, green boxes enclose the functionally characterized genes in their respective pathways.

Brassinosteroids (BR) comprise an important group of steroidal hormones originally isolated from the pollen of brassica plants (Rehman et al., 2022a). Brassinolide (BL) is the biologically active BR that is synthesized from compound campesterol with the aid of a cytochrome P450-mediated pathway (Bishop, 2007). This was a relatively novel and less studied hormone in the past, but recently it has gained attention as an active growth-promoting hormone owing to its involvement in many physiological functions (Fridman and Savaldi-Goldstein, 2013; Rehman et al., 2022b). Several genes are involved in the signaling pathways of brassinolide from BL perception to activation of responsive genes for example receptor-like kinase BRASSINOSTEROID-INSENSITIVE 1 (*BRI1*), BRI1-ASSOCIATED RECEPTOR KINASE 1, SOMATIC EMBROGENESIS RECEPTOR KINASE 1, and a repressor gene GSK3-like kinase BIN2 (BRASSINOSTEROID-INSENSITIVE 2) (Sánchez-Rodríguez et al., 2010). After the discovery of BL, mutant analysis in *Arabidopsis* revealed that plants deficient in the genes related to BL biosynthesis or signaling pathways showed dwarf phenotype, compromised male fertility, delay in flowering time, altered patterns of vascular development, and impaired photomorphogenesis (Feldmann et al., 1989). In a very recent study on maize, an endoplasmic reticulum localized gene, i.e., *ZmD11* related to the biosynthesis of BL rescued the panicle architecture and plant height in *cpb1* mutant in maize and rice. *ZmD11* increased seed length, seed weight, and both seed starch and protein contents in rice and maize crops (Sun et al., 2021). brassinosteroid-deficient dwarf1 *(brd1)* gene encoding brassinosteroid C-6 oxidase having a maize *lilliputian1* allele *(lil-1)* caused alteration in gravitropic response of root, leaf cell density, and more wax deposition conferring the adaptive mechanism to stress (Castorina et al., 2018). BR receptor, i.e., BRASSINOSTEROID INSENSITIVE1 (*BRI1*) RNA interference (RNAi) knock-out mutants in maize showed overall dwarf stature, shortened internodes, folded dark green leaves, decreased auricle formation, and feminization of male flowers (Kir et al., 2015). Similarly, in sorghum, the nuclear localization of BRASSINOSTEROID INSENSITIVE 2 (*BIN2*) was inhibited by DW1 indicating its role in BR signaling. Sorghum lines harboring mutated Dw1 (dw1) showed impaired skotomorphogenesis, lamina joint bending, and insensitive to BR gene regulation and feedback (Hirano et al., 2017) (Figure 1A).

This cluster of genes involved in biosynthesis and signaling of the important growth-promoting hormones highlights the connections with the cell wall, carbohydrates, and photosynthesis-related pathways. Further studies need to elucidate growth patterns of double or triple mutants from multiple hormone pathways at transcriptional and biochemical levels for efficient biomass response.

#### Cell Wall-Related Genes

The cell wall is a non-living protective cell layer that comprises 70% of the world’s plant biomass (Poorter and Villar, 1997; Pauly and Keegstra, 2008). Second-generation cellulosic biofuel (bioethanol, biohydrogen, and biomethanol) produced from the plant biomass mostly comes from the cell wall. During plant growth and cell extensibility, several processes are involved among which cell wall loosening and rearrangement strongly contribute in plant biomass traits. In plant species, numerous gene families related to cell wall biogenesis, membrane trafficking, remodeling, secondary cell wall synthesis, and signaling comprise ∼10% of plant genomes (Lauter et al., 2005; Yong et al., 2005; McCann and Carpita, 2007).

Many studies involving plant biomass engineering techniques showed a strong effect on cell wall-related genes on growth and biomass accumulation processes in C_4_ biofuel crops e.g., miscanthus (van der Weijde et al., 2013), sorghum (Scully et al., 2016; Xia et al., 2018; Tetreault et al., 2020), and sugarcane (Jung et al., 2012; Bottcher et al., 2013). Genes responsible for cellulose synthesis mainly include members of cellulose synthase (*CesA*) and cellulose synthase-like (*Csl*) families. A recently published comparative study have identified 77, 35, and 109 CesA/Csl genes in *Miscanthus floridulus*, *S. bicolor*, and *S. spontaneum*, respectively. Among the 10 groups of CesA genes classified by phylogenetic approaches, a new group was identified in *Miscanthus floridulus*, i.e., *CesAX* which was not present in C_3_ rice. Higher expression of CesA genes and their duplicates mainly followed by WGD (Whole Genome Duplication) showed the additive effects in gene expression levels resulting in more cellulose accumulation (Zhang et al., 2021). Silencing or mutations of *CesA* genes in *Arabidopsis* and certain other monocots have resulted in certain functional deformities but there is no authentic evidence that over-expression of *CesA* will certainly increase the cellulose content of the cell wall. In *Miscanthus* × *giganteus*, cloning of six *MgCesA* genes showed the involvement of MgCesA2, 3, 4, 7, and 8 in primary cell wall biosynthesis and rest in (MgCesA10, 11, 12) secondary cell wall synthesis and formation of cellulose synthase complex (Zeng et al., 2020).

Two cellulose interacting genes *TED6* and *TED7* enhanced the cellulose synthesis in xylem vessel elements, and lack of function mutants resulted in the failed secondary cell wall formation in *Arabidopsis* (Endo et al., 2009). Similarly, expression profiling of interspecific sugarcane hybrids showed upregulation of *CesA*, laccases, and callose synthase-related genes in high biomass extreme F_2_ segregants (Wai et al., 2017). Lignin has been an undesirable component of the cell walls in terms of bioenergy generation, although it accounts for ∼30% terrestrial organic carbon fixation in the biosphere (Battle et al., 2000). In sorghum, brown midrib mutant (*bmr*) showed decreased levels of lignin and the formation of an altered subunit. The *Bmr* gene is a biosynthesis gene in monolignol units, which yield the hydroxycinnamic subunits of lignin. Another class of genes, laccases, are involved in the oxidation of monolignol units before the incorporation into cell wall polymers (Bonawitz and Chapple, 2010). *In vitro* oxidation of lignin precursors (Liang et al., 2006) and localization in lignin synthesizing tissues (Ranocha et al., 2002; Caparrós-Ruiz et al., 2006) have been experimentally proved by laccases in plants. In *Arabidopsis*, T-DNA insertion lines exhibited the reduced lignin content in single mutants of *AtLAC4* and *AtLAC17* whereas, double mutants displayed 40% reduced lignin but with irregular xylem tissues (Berthet et al., 2011). Sugarcane, a benchmark of biomass-derived biofuels showed the strong interactions of *SofLAC* genes with phenylpropanoid biosynthesis genes in a co-expression network. For the confirmation of monolignols oxidation, complementation of the *SofLAC* gene under the native promoter *AtLAC17* was performed in *Arabidopsis lac17* mutants having reduced lignin. SofLAC repaired the lignin content in *Arabidopsis* but lignin composition was altered in complemented *lac17* mutant lines (Cesarino et al., 2013). Xyloglucans (XyG) comprise a major class in hemicellulose proportion of cell wall and are extensively found in primary cell walls of eudicots and non-gramineous monocots. In *Arabidopsis*, double mutant *xxt1 xxt2* displayed a considerable reduction in detectable xyloglucan and altered mechanical properties (Cavalier et al., 2008). For an ideal bioenergy crop, higher lignin content is an undesirable trait due to its recalcitrance to degradation, whereas higher crystalline cellulose content is favored due to its digestibility. Conversely, hemicelluloses are crosslinking both lignin and cellulose causing a decrease in cellulose crystallinity, but a reduced level of hemicellulose branching ensures easy separation of cell wall components (Torres et al., 2015). However, molecular alteration of hemicellulose is handicapped due to its vague and complex biosynthesis and subsequent pathways. Research advances to this field are nevertheless confined at molecular levels in model species whereas, application of this knowledge in bioenergy-related species is the main goal (Figure 1B).

#### Transcriptional Regulation and miRNA Role

Transcription factors are the genes encoding proteins (besides RNA polymerase) that are essentially required for transcription. Owing to the regulatory role in transcription activity, they are capable of controlling the expression of many downstream key genes related to growth and development. In plants, DNA transcription involves more than 1,500 TFs to regulate target genes by binding with cis-regulatory elements in the promoter region (Singh et al., 2002). Secondary cell wall formed between the primary cell wall and cell membrane strongly contributes to the development and is an important attribute for the biofuel industry. Biosynthesis and remodeling of cell wall components are achieved through an orchestrated action of TFs and downstream genes. Therefore, detailed knowledge of transcription factors controlling secondary cell wall initiation genes, polysaccharides synthesis, lignification process, and a parallel process of programmed cell death (PCD) of xylem cells (Ohashi-Ito et al., 2010) is important to dissect for biomass regulation (Table 1).

**FIGURE 2:**
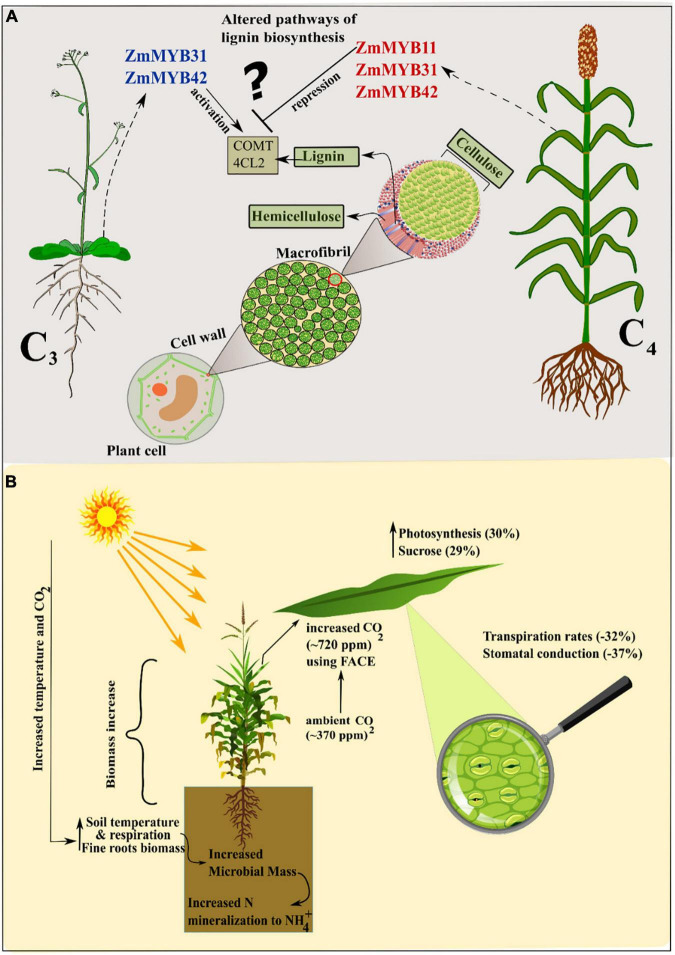
**(A)** Contrasting transcriptional regulation patterns of *MYB* TFs in C_3_ and C_4_ plants. In C_3_ (*Arabidopsis*) ectopic expression of maize TFs enhance lignin biosynthesis whereas in C_4_ (Maize) lignin content is reduced. This gives rise to lineage-specific transcription in C_4_ plants. **(B)** Increase in temperature and CO_2_ enhances photosynthesis and altered control of stomatal aperture enhancing WUE. Soil microbiota mass in the rhizosphere is also increasingly responsible for modifications in the nutrient pool.

Transcription factors of NAC (NAM—No Apical Meristem, ATAF, CUC—CUP/SHAPED Cotyledon) family activates a nexus of downstream transcription factors for example *MYBR2B3*, and act as master switches by binding with cell wall biosynthetic genes (Martins et al., 2018). SHINE “SHINE/WAX INDUCER” (*SHN/WIN*) TF is a member of ETHYLENE RESPONSIVE FACTOR (*ERF*) that functions as a regulator of cell wall biosynthesis genes, resulting in increased cellulose and decreased lignin contents (Ambavaram et al., 2011). In switchgrass (*Panicum virgatum*) *PvERF001* gene which is the homolog of *AtSHN2* conferred activation of cell wall synthesis and accumulation of biomass (Wuddineh et al., 2015). Likewise, sugarcane transcription factor *ShSHN1* overexpressed in rice induced changes in cell wall composition and increase in biomass by (91–340%), pectin (26–209%), cellulose content (10–22%), and saccharification efficiency (5–53%) in rice transgenic plants (Martins et al., 2018). McCarthy reported that *AtMYB46* and its paralog *AtMYB83* are found to function as activators of the secondary cell walls and are expressed in xylem fibers and vessels during secondary cell wall development (McCarthy et al., 2009). In *Arabidopsis myb46/83* double mutant, maize orthologs of *AtMYB46/83* successfully complemented the secondary cell wall synthesis and thickening after rescuing the defected walls (Zhong et al., 2011). Similarly, in switchgrass, overexpression transgenic lines of R2R3-MYB (*PvMYB4*) showed increased biomass up to ∼63% and reduced lignin content around 50% (Shen et al., 2012). Some MYB genes in grasses, e.g., maize and switchgrass function in lineage-specific fashion regarding lignin biosynthesis regulation (Agarwal et al., 2016). For example, co-immunoprecipitation and ChIP-seq assays (co-IP) assays showed that *ZmMYB11*, *ZmMYB31*, and *ZmMYB42* induced reduction in expression of lignin biosynthesis-related genes *COMT* (caffeic acid-O-methyltransferase) and *4CL2* in maize (Vélez-Bermúdez et al., 2015). ZmMYB31 and ZmMYB42 in exogenous expression in *Arabidopsis* redirected phenylpropanoid and lignin-related genes in *Arabidopsis* contrary to maize. Moreover, *ZmMYB31* and *ZmMYB42* didn’t down regulate the *ZmF5H* (ferulate-5-hydroxylase) gene in maize as compared to *Arabidopsis*, leading to a decrease in S/G ratio (S, syringl units; G, guaiacyl units) (Sonbol et al., 2009; Fornalé et al., 2010; Vélez-Bermúdez et al., 2015). Ectopic expression of transcription factor *SbMyb60* in sorghum showed involvement in monolignol biosynthesis pathways and increased lignin concentration and plant biomass. Constitutively overexpressing *SbMyb60* displayed enhanced lignification in leaf midribs and soluble phenolic compounds in plant biomass (Scully et al., 2016). This suggests that in monocot grasses the route of MYB TFs and their regulatory pathways are more diverse and need to be investigated considering the models from grasses. Furthermore, the differences in C_3_ and C_4_ photosynthetic regulatory pathways should be studied in detail to increase the cellulose and hemicellulose contents and decreased contents of recalcitrant, i.e., lignin. (Figure 2B). Finger millet transgenic plants over-expressing *bHLH57 (BASIC* HELIX-LOOP-HELIX) depicted resistance to salinity stress with enhanced photosynthetic efficiency and increased biomass (Babitha et al., 2015). *AHL* (AT-HOOK MOTIF NUCLEAR LOCALIZED) family of transcription factors in *Arabidopsis* controls the petiole and rosette growth and architecture by antagonizing the role of growth-promoting PHYTOCHROMEINTERACTING FACTORS (*PIFs*) (Favero et al., 2020). Maize zinc finger protein *Dof1* transcription factor increased the nitrogen use efficiency in transgenic sorghum and wheat. Tissue-specific expression of *ZmDof 1* under rbcS1(maize) promoter increased growth by activation of carbon skeleton metabolism, i.e., *PEPC* activity (Peña et al., 2017) (Figure 1C).

SQUAMOSA Promoter-Binding Protein-Like (*SPL*) transcription factors are regulated by microRNAs (miRNA), i.e., miR156 and AtSPL9 which positively regulate another miRNA miR172 (Chen et al., 2010). *AtSPL9* TF binds to the promoter region of *the MIR172* gene and induces the transcription activity of downstream genes and repress adult-related characteristics in *Arabidopsis*. At the later plant stage, *the miR156-AtSPL9-miR172* regulatory pathway progresses with the decrease in *miR156* levels and increase in *miR172* leading to repression of FLOWERING LOCUS T (FT) gene. This event allows entering the plant to the reproductive phase and the same regulatory pathway is conserved in maize where *miR172* represses *Glossy15*, an *AP2-like* TF (Lauter et al., 2005; Chuck et al., 2007). This interactive regulatory role of transcription factor and microRNA is an effective molecular tool to extend the vegetative phase for enhanced sink capacity and biomass accumulation. Dumitrache et al. (2017) developed independently overexpressing (OE) and silenced (KD) transgenic switchgrass lines of specific genes and *miRNA GAUT4-KD*, *miRNA156-OE*, *MYB4-OE*, *COMT-KD*, and FPGS-KD (Dumitrache et al., 2017). Continuous monitoring of 2-year ratoon transgenes showed increased contents of carbohydrates by 12% and ethanol yields by 21% as compared to controlled conditions. In *Arabidopsis*, the use of zinc finger artificial transcription factor (*ZF-ATF*)-mediated interrogation lines helped in understanding growth and biomass-related characteristics. Introgression lines of two *Arabidopsis* genomes harboring 3F-EAR encoding T-DNA and 3F-VP16 encoding T-DNA constructs showed substantially large phenotypes. Whereas, 3F-EAR is *Arabidopsis* based ERF-associated Amphiphilic Repression (*EAR*) motif and acts as a dominant repressor evident from the previous studies and VP16 protein originated from the herpes simplex virus as a transcriptional activator (Ohta et al., 2001; Hiratsu et al., 2003; McCarthy et al., 2009). Further research is needed to elucidate the differences in transcriptional regulation of *Arabidopsis* and C_4_ grasses, involving the promoter analysis to identify important cis-elements and the spatio-temporal regulation of TFs, affecting development-related genes.

#### Hybridity and Polyploidy

Hybrids and polyploids are common in plants. Hybridization between and within species is a natural process and is estimated to occur in ∼25% of plant species (Mallet, 2005). Hybrid vigor is a common consequence of hybridization and refers to superior hybrid performance in yield, biomass, or other agronomic parameters. Polyploidy refers to a cell or organism having two or more sets of basic chromosomes. An autopolyploid is derived from genome duplication within the same species, such as alfalfa (*Medicago sativa*), sugarcane (*Saccharum*), and potato (*Solanum tuberosum*), while allopolyploids are formed by chromosome doubling following hybridization between species. Allopolyploid is a “doubled interspecific hybrid,” which leads to heterozygosity and hybrid vigor fixation. Many crops like cotton (*Gossypium hirsutum*), bread wheat (*Triticum aestivum*), and oilseed rape (*Brassica napus*) are cultivated as allopolyploids while rice (*Oryza sativa*), and maize (*Zea mays*) are mainly grown as hybrids. Both ploidy and hybridity affect growth vigor and cell size which are directly associated with plant biomass production (Chen, 2010).

In maize, increased ploidy had a detrimental effect on plant size which increases from haploid to triploid, but reduces in tetraploid (Riddle et al., 2006), and is consistent with smaller haploid *Arabidopsis* plants than diploids (Ravi and Chan, 2010). Induced polyploidy in hybrids may facilitate improving yield components, diminish hybrid vigor breaks in subsequent generations and restore inter-subspecific hybrids fertility (Miller et al., 2012). In *sorghum*, the colchicine-treated polyploidy induced plants showed high biomass with longer leaf length and stronger root system (Ardabili et al., 2015). A triploid *Miscanthus × giganteus*, C_4_ grass is considered an excellent bioenergy crop due to its capacity to capture greenhouse gases by sequestering carbon in underground rhizomes and high biomass production when compared to diploid Miscanthus species (Chae et al., 2013). The modern sugarcanes are polyploid interspecific hybrids combining disease resistance, hardiness, and ratooning of *Saccharum spontaneum* and high sugar content from *Saccharum officinarum*. Genome restructuring and gene expression modifications in these cultivars due to polyploidy provide a selective advantage for a wider geographical adaptation, increased vigor, sucrose, and fiber content (Ming et al., 2001; Hoang et al., 2015). There is increased global demand for alternative fuel sources, and sugarcane is gaining importance as a biofuel crop with its high biomass production potential, besides being a major sugar crop.

## Environmental Cues Influencing Biomass Accumulation

Plants being sessile in nature are exposed to constantly changing environment enveloping around them. In the presence of judicious input resources and ideal genotypes, still plants are exposed to fluctuating environment in terms of CO_2_ concentration, irradiance, and temperature which are function of plant growth. Here we reviewed that how fluctuations in external environment alter gene expression of important pathways and eventually biomass accumulation patterns.

### Ambient CO_2_ Fluctuations

With the gradual increase of GHGs in the atmosphere, the extent of CO_2_ is also increasing in the air as CO_2_ also comes under the category of greenhouse gases. This increase in GHGs tends to rise the global temperature which seriously changes the genetic and physiological attributes affecting the growing patterns of plants. This global warming accompanied by climate change has increased the variability of precipitation and a continuous increase in ambient CO_2_ concentration up to 490–1,260 ppm by the end of the twenty-first century (IPCC, 2007). Considering the global changes, fluctuations in CO_2_, light, and temperature are having both direct and indirect effects on the growth and biomass production of C_3_, C_4_, and CAM photosynthesis plants (Ainsworth and Long, 2005). Owing to anatomical and functional modifications in C_4_ species, it was assumed that C_4_ plants will be less affected by CO_2_ rise as ambient CO_2_ already meets the maximum saturation due to bundle sheath cells, as for C_3_ plants CO_2_ is a limiting factor for maximum photosynthesis. However, previous researches suggest different reasons for the increased response in terms of growth and productivity against elevated CO_2_ in C_4_ plants as (1) direct effect on Rubisco as CO_2_ saturation point increases (Ziska and Bunce, 1997), (2) leakiness of bundle sheath cells (Watling et al., 2000), (3) young leaves are supposed to undergo preliminary C_3_ photosynthesis system (Cousins and Bloom, 2003), (4) reduced stomatal aperture to enhance WUE or maintaining inner optimal temperature (Ghannoum et al., 2000).

Dieleman et al. (2012) published a meta-analysis of multifactorial experiments in which all treatments showed that increased CO_2_ and warming increased plant biomass and soil respiration. An increase in only CO_2_ treatment elicited more biomass of fine roots, soil respiration, and a decrease in foliar nitrogen (Dieleman et al., 2012). In another study De Souza et al. (2008) compared the effects of ambient (∼370 ppm) and increased (∼720 ppm) CO_2_ concentration on sugarcane growth and biomass. Elevated CO_2_ led to an increase of 17% in plant height, 30% in photosynthesis, 29% in sucrose contents, and 40% more accumulation of plant biomass (De Souza et al., 2008). This is thought to be achieved by physiological modifications regarding WUE as transpiration rates and stomatal conduction was reduced by –32 and –37%. An experiment involving free-air carbon enrichment (FACE) showed an elevated photosynthetic rate in young leaves and increased biomass and leaf number in sorghum and maize, respectively (Maroco et al., 1999; Cousins and Bloom, 2003). Recently, a study focused on the membrane properties and photosystem II activity of maize and pearl millet under elevated CO_2_ and temperature. The results showed that maize outperformed in biomass accumulation in the presence of high CO_2_ while pearl millet was more responsive in high temperature (Bordignon et al., 2019). Conversely, a comparative study conducted on two weedy species of C_3_ (*Chenopodium album*) and C_4_ (*Setaria viridis*) plants showed the decreased biomass at elevated temperature alone but a dramatic increase in biomass and seed yield by 33.9 and 114.4%, respectively, at increased temperature and CO_2_ concentrations in *Chenopodium album*. On the other hand, *Setaria viridis* showed 1.6- and 1.3-fold more biomass in an increased temperature and CO_2_ conditions as compared to control and only increased temperature (Lee, 2011). Experiments conducted on wheat and maize as the representatives of C_3_ and C_4_ plants showed that high CO_2_ in C_3_ (wheat) helps in ammonium NH_4_^+^ assimilation which was very less in ambient CO_2_ and showed declined rate of photosynthesis. Overall results showed that cellular and chloroplast CO_2_ enhanced electron flux in wheat as compared to maize. Several experiments showed that C_3_ plants are more responsive toward elevated CO_2_ as they reduce the extent of photorespiration and benefit more from enhanced CO_2_. However, recently reported from a 20-year continuous investigation using FACE experiment on 88 grassland plots, that for 12 years C_3_ plants showed increased biomass owing to higher eCO_2_ (Equivalent CO_2_) (Reich et al., 2018). Whereas, in subsequent 8 years a shift in this trend was seen from C_3_ to C_4_ plants, which resulted in enhanced biomass and soil nitrogen mineralization in C_4_ plants. These results are challenging to current concepts regarding the C_3_-C_4_ eCO_2_ paradigm (Figure 2A).

### Circadian Rhythm Modulations by Environmental Factors

The instabilities of external cues of the environment, such as light, temperature, and nutrition, evoke a well-developed endogenous time-keeping mechanism in plants which is called the circadian cycle that allow in the modulation of energy and developmental metabolism. For example, fluctuations in the diurnal rhythm of light duration, temperature, and nutrition level modify intricate transcriptional and post-transcriptional loops in plants similar to animals (Harmer et al., 2001; Inoue et al., 2017). In *Arabidopsis*, the circadian clock starts by the mutualistic interaction of two transcription factors circadian clock associated 1 (*CCA1*) and late elongated hypocotyl (*LHY*) (Mizoguchi et al., 2002) during the morning hours. These two MYB TFs (*CCA1, LHY*) repress the transcription of TIMING OF CAB EXPRESSION 1 (*TOC1*) also known as PSEUDORESPONSE REGULATOR 1 (*PRR1*), which acts as a regulator of many downstream genes. *CCA1* Hiking Expedition (*CHE*) (Pruneda-Paz et al., 2009), and GIGANTEA (*GI*) (Strayer et al., 2000) also acts as a positive regulator in circadian loops, whereas, *CCA1* and *LHY* mRNA decrease during the mid-day by *TOC1* homologs (*PRR9* and *PRR7*) in feedback mechanism (Farré et al., 2005). During the evening, Lux Arrythmo (LUX) mediated transcriptional repression of PRR9 takes place by early flowering 4 (*ELF4*) and (*ELF3*) (McClung, 2014).

In grasses, growth activity is mediated by a rib zone called shoot apical meristem and is strongly influenced by light and shade conditions. In densely grown sorghum populations, leaves experience more shade conditions and inhibit shoot branching, more stem elongation, and early flowering. A combination of these responses is called Shade Avoidance Syndrome (SAS) (Casal, 2013), which is a survival strategy in plants for the quest for more sunlight and resources. Plant photoreceptors act to monitor the light environment and perception, and they work in synchrony with the circadian clock to regulate growth and development in plants (Devlin and Kay, 2001). Fluctuations in the light intensity change the expression of 24 circadian genes in bioenergy sorghum, among which CCA1 and LHY showed 12.7-fold lower and 5.9-fold higher expression in internodes of shade-treated plants in comparison to control. CCA1 and LHY regulate the expression of downstream genes for example in sorghum, the homolog of Arabidopsis Granule Bound Starch Synthase1 (*GBSS1*) gene which functions in starch biosynthesis was also down regulated by 6.6-fold. Other clock-related genes for example evening core clock genes were upregulated in shade-exposed plants. For example, the expression of *BT2*, and THIAMIN C SYNTHASE (*THIC*) function in important pathways related to hormones, sugars, and thianmin synthesis are regulated by circadian cycle genes (Mandadi et al., 2009). Similarly, 6 days of exposure to extended darkness resulted in malfunctioning in photosynthetic pigments, reduction in photoassimilates, and total soluble and insoluble carbohydrates in maize. However, CO_2_ exchange was not disturbed but transient carbon pools were largely consumed by elevated levels of nocturnal respiration rather than transport toward the sink. Underlying processes may involve signals by trehalose-6-phosphate and circadian rhythms which are controlling stress response in multi-dynamic pathways (Graf et al., 2010; Wingler et al., 2012). Light quality also mediates growth responses by genetic and physiological modifications. Transcriptome and metabolome profile of blue light exposed maize plants showed genes and metabolites related to stomatal, carotenoid, photosynthesis, and circadian cycle-related genes. *CCA1* gene was upregulated in presence of blue light, this upregulation is consistent with the expression of many downstream genes related to photosynthesis, starch synthesis, and stomatal development processes (Liu and Zhang, 2021). In maize, the early stage of light exposure stimulates the circadian rhythm genes to increase light absorbance by regulation of photosynthetic genes (Khan et al., 2010). As C_4_ plants possess well-developed Kranz anatomy for the specialized storage of CO_2_ and water. In maize plant *ZmPIP4c*, a water transport aquaporin gene showed high expression profiles in bundle sheath cells during diurnal change and is potentially responsible for the transport of water in mesophyll cells. The synchronization in the expression of NAD-malic enzyme gene (*NAD-ME*), phosphoenolpyruvate carboxylase gene (*PEPC*), and carbonic anhydrase gene (*CA*) from base to tip is consistent with the circadian rhythm regulating cycle for efficient WUE in both light and dark conditions (Xiang et al., 2020). Moreover, as already discussed hybridization leads to heterosis, which is the outcome of enhanced photosynthesis and metabolism possibly influenced by CIRCADIAN CLOCK ASSOCIATED1 (*CCA1*). Two homologs of maize, *ZmCCA1a*, and *ZmCCA1b* are diurnally upregulated in *Arabidopsis*, *cca1 Arabidopsis* mutant was complemented by *ZmCCA1*. Whereas, *ZmCCA1b* showed disruption in circadian rhythms leading to reduced heterosis and plant height in the greenhouse and slightly compensated in field conditions upon light exposure. In hybrids, the temporal shift of *ZmCCA1*-binding targets suggest the activated photosynthesis and growth vigor genes in the morning phase relative to the inbred lines (Ko et al., 2016). The behavior of circadian genes is a good indicator to enhance our understanding of environment-influenced genetic modulations.

## Tools and Strategies to Enhance Biomass

### Hybridization and Molecular Techniques

Until now, some plant species have been given attention for the improvement of biomass which includes miscanthus, switchgrass, willow, poplar, and eucalyptus, with their improvement history dating back to the second half of the twentieth century (Allwright and Taylor, 2016; Clifton-Brown et al., 2019). Their improvement relied on hybridization or breeding methods (crossing of different strains, species, or lines) leading to heterosis or hybrid vigor of the F_1_ heterozygotes with higher fitness in the population. Heterotic fitness refers to superior growth, stature, fertility, and biomass in offsprings. Several factors, for example, transcriptional regulation and epigenetic changes drive improved characters in hybrids. We have already reviewed that breeding techniques are employed mostly in C_4_ grasses for achieving enhanced biomass. However, the approval and release of commercial cultivars take a long period of time, which factually delays the cultivation in agriculture systems and slows down the progress of conventional breeding (Clifton-Brown et al., 2019). Conventional breeding techniques advance our understanding toward marker-assisted selection for biomass-related traits, stress tolerance, and scarification in biofuel grasses and woody plants. In switchgrass, marker-assisted breeding enabled the understanding of substitution of cell wall hemicellulose polymers backbone and remodeling (De Souza et al., 2015) whereas identification of specific loci was identified from potential markers for high ethanol generation from switchgrass populations (Chen et al., 2016). Prairie cordgrass is a potential C_4_ bioenergy crop, and two clones of prairie cordgrass were crossed and developed SSR markers for marker-assisted selection of biomass traits (Gedye et al., 2012). Another study comprising 28 sugarcane genotypes identified simple sequence repeat (SSR) markers associated with stalk number and stalk volume (Bilal et al., 2015). In another experiment, 40 putative quantitative trait alleles (QTAs) were identified from a self-crossed (295) population of “R570,” with each allele contributing to phenotypic variation by 3–7% in sugarcane (Hoarau et al., 2002). Likewise, in sorghum, four QTLs were identified that control tiller number and formation (Hart et al., 2001). Recently research on the genotypes of *M. sinensis* indicated the genetic diversity of cell wall constituents and concluded that a higher ratio of para-coumaric acid to lignin contents and trans-ferulic acid (TFA) increased the saccharification efficacy (van der Weijde et al., 2017a). In sugarcane, stalk number is influenced by genes and their alleles with additive and non-additive effects or their interactions (Hongkai et al., 2009; Carvalho-Netto et al., 2014). Considering the use of genome editing techniques for bioenergy crops, the use of Transcription activator-like effector nucleases (TALENs) have been employed for targeted modification in the genome. High lignin content is an undesirable character for biofuel crops as in sugarcane, TALEN induced mutation in caffeic acid O-methyltransferase (*COMT*) sequences modified cell wall compositions. Pyrosequencing showed mutation frequencies up to 99% and revealed 29–32% reduced lignin and elevated hemicellulose contents (Jung and Altpeter, 2016). Similarly, RNAi-derived *COMT* silenced sugarcane callus-derived plants showed a 12% reduction in lignin and 32% improved scarification but compromised agronomic performance (Jung et al., 2013). CRISPR/Cas9 is a recent genome-editing technique expanding its applications to construct desirable genetic circuits (Khakhar et al., 2018). CRISPR/Cas9 system was employed in switchgrass for the reduction of lignin contents (Park et al., 2017). Knock-out mutant of the *Pv4CL1* gene encoding 4-coumarate: coenzyme A ligase (4CL) displayed increased scarification as compared to wild. Tiller formation is one of the indices of biomass, two genes grassy tiller1 (*gt1*) and teosinte branched1 (*tb1*) control tiller formation in maize (Whipple et al., 2011). BRANCHED1 (BRC1) gene in sorghum is the homolog of *tb1* gene, ectopic expression of *tb1* gene in *Arabidopsis* promoted axillary buds formation *Arabidopsis* (Kebrom et al., 2006). Genome editing-based mutagenesis using CRISPR/Cas9 showed proliferated tillers in switchgrass as compared to wild plants (Clifton-Brown et al., 2019). In sorghum, *by1* mutant obtained by knocking out BIOMASS YIELD 1 gene using CRISPR/Cas9 displayed reduced plant height narrow stem length, erect and narrow leaves, and abnormal floral organs. BIOMASS YIELD 1 gene translates into an enzymatic protein catalyzing the first step of the shikimate pathway (Chen et al., 2020). *BY1* gene showed its role in primary metabolism and secondary metabolites for example flavonoids. In *Arabidopsis* hormone activated Cas9-based repressor (*HACRs*) showed significant results that can be utilized to achieve high grass biomass and economic yield (Khakhar et al., 2018). Similarly, in sugarcane, using transgenic and molecular techniques, an ortholog of the *SLR1/D8/RHT1/GAI* gene showed substantial stem growth and structural modifications in storage organs by regulating source-sink allocation changes (Garcia Tavares et al., 2018). Although several transgenic lines with enhanced features as bioenergy crops have been developed, there is a need for a suitable selection process and quality evaluation. However, due to cross-pollination in many grass species, transgenic lines pose a threat of seed contamination. Introgression and hybridization require labor-intensive and time-consuming efforts with uncertain outcomes. Therefore, recent genome editing techniques for example synthetic genetic circuits (SGC) or CRISPR offer sophisticated and foreseeable mutation induction in first-generation mutant lines (Scheben and Edwards, 2018). Moreover, Near-infrared spectroscopy (NIRS) and thermal aerial imaging technologies aid the phenotyping of constituents and high-throughput options to screen abiotic stress tolerance, respectively (van der Weijde et al., 2017b). Few examples in Table 2 highlight the use of integrated phenotyping and molecular technologies for biomass related research.

**TABLE 2:** Few examples of genome editing techniques, engineering the C_4_ plants for biofuels.

Crops	Targeted genes	Technique	Improved traits	Associated pathway	References
Sugarcane	*COMT*	Transcription activator-like effector nucleases (TALENs)	11–32% reduced lignin Increased hemicellulose contents	Methyltransferase cell wall	Jung and Altpeter, 2016
Sugarcane	*COMT*	RNAi	12% reduction in lignin and improved scarification by 32%	Cell wall	Jung et al., 2013
Switchgrass	*Pv4CL1*	CRISPR/Cas9	8–30% reduced lignin 7–11% and 23–32% increase in glucose and xylose release	Lignin synthesis pathways	Park et al., 2017
Sorghum	BIOMASS YIELD 1 BY1	CRISPR/Cas9	Displayed reduced plant height, narrow stems, erect and narrow leaves, and abnormal floral organs.	Shikimate pathway	Chen et al., 2020

### Nitrogen Management

Plant biomass of crops including C_4_ plants is influenced by a variety of variables, i.e., plant genotype, photoperiod, solar radiation, soil temperature, soil humidity, and many more. Soil nutrient availability is one of the most significant variables determining the crop biomass in C_4_ crops. So, by regulating the optimal amounts of nutrient availability in soil, growers may optimize the biomass output for biofuels and of course economic gain (grain production). Soil degradation and low soil fertility status significantly minimize the nutrient availability in the soil to plants (Chatzistathis and Therios, 2013; Tanveer et al., 2019). Optimum fertilization appears to be the most common method chosen by farmers in instances of restricted nutrient availability in soils to improve the biomass of cultivated C4 crops. Macronutrients, i.e., nitrogen, phosphorous, magnesium, potassium, sulfur, and calcium, and micronutrients, i.e., copper, zinc, manganese, iron, chlorine, and molybdenum are classified as important nutrients for improving the biomass of C_4_ crops. Nutrient scarcity has a detrimental impact on biomass productivity (Anjum et al., 2019). Vegetation flushes of C_4_ crops are severely hampered by nitrogen shortage as nitrogen is an integral component of chlorophyll, pyrimidines, purines, amino acids, proteins, and nucleic acids in C_4_ crops. Borges et al. (2019) reported that the appropriate method of nitrogen application at the appropriate time (early season) significantly improved the biomass of sugarcane (*Saccharum officinarum*) by 30% as compared to traditional fertilizers practices. In addition, climate prediction models guided nitrogen management methods can be opted, as the climate is a significant determinant of crop growth, nitrogen demand, and nitrogen losses processes. Seasonal climatic projections might be used to establish nitrogen management plans for “dry” and “wet” years, directing application rate, timing, and frequency of nitrogen fertilizer application, as well as the advantages of employing different types of nitrogen fertilizer in C_4_ crops like sugarcane, maize (*Zea mays*), sorghum (*Sorghum bicolor*), pearl millet (*Pennisetum glaucum*), and Napier grass (*Pennisetum purpureum*) (Anjum et al., 2019). Moreover, Khan Khyber et al. (2010) reported that integrated application of 50% urea with 50% poultry manure significantly enhanced the grain yield of maize by 57.14%, respectively, as compared to plots having 0% nitrogen application. Although much effort has previously been done to maximize yields while maintaining high nutrient utilization efficiencies, more integrated approach results are still needed to minimize the nitrogen inputs while maintaining optimum usage efficiency in C_4_ crops (Noor, 2017). To attain the maximum production of biomass in C_4_ crops, maintaining the optimum levels of all the necessary soil nutrients should always be taken care of. However, determining the best nutrient prescription in terms of boosting productivity especially biomass production in C_4_ crops while also guaranteeing food security and environmental friendliness is a difficult task, and still needed further studies and detailed analysis.

### Silicon Foliar Application

Silicon is a chemical element having atomic number 14 and is represented with the symbol Si. In plants application of silicon significantly enhances the crop biomass; improves the tolerance to biotic and abiotic stresses, and aid plant stability and protection (Zargar et al., 2019). In connection to the enhancement of cell wall elasticity and stiffness, silicon that is firmly linked to the cell walls is naturally present as a structural material. When the quantity of monosilicic acid in the xylem sap is high, it becomes a significant osmolyte, increasing the plant’s water and osmotic potential. Furthermore, in terms of structural material and osmolytes, Si consumes less energy than biomolecules like proline and lignin. As a result, for a cheap cost, silicon can enhance the homeostasis of C_4_ plants’ tolerance to a variety of biotic and abiotic stressors in terrestrial environments. C_4_ plant biomass recovery mediated by silicon is thought to have a bell-shaped response curve to abiotic stressors and an S-shaped response curve to biotic stresses. Silicon treatment to abiotic and biotic stressed crops can boost averaged plant biomass carbon and crop productivity by 35 and 24%, respectively. The efficacy of silicon-mediated restoration, on the other hand, varies substantially depending on the plant species and cultivars, the severity of abiotic and biotic stressors, and the amount of bio-available silicon. Ashraf et al. reported that the application of silicon significantly improved the biomass production in sugarcane by 77% under salinity stress (Ashraf et al., 2009). Similarly, the application of calcium silicate improved the crop biomass of sugarcane and enhanced the resistance in sugarcane against stem borer (Keeping and Meyer, 2002; Meyer and Keeping, 2005). A study conducted on maize reported that the application of silicon under water stress conditions significantly enhanced the crop biomass and nutrient uptake (Kaya et al., 2007). It was reported that the application of silicon significantly improved the plant biomass under agricultural soil contaminated with heavy metals like cadmium (Liang et al., 2005; Lukačová et al., 2013), and in arsenic (Ullah et al., 2016). Application of silicon is a significant option for improving the crop biomass of C_4_ crops, but still more research and detailed analysis should be done as most of the silicon application trials have so far been done in pots, field-scale to eco-system-scale investigation is needed. Moreover, several issues, such as the coupling relations between Si and plant essential elements, the efficiencies of Si-mediated plant biomass carbon restoration among plant species and stress intensities, and the relationship between the biogeochemical Si cycle and the resilience of terrestrial ecosystems, all require more research, particularly in fragmented landscapes.

### Foliar Application of Plant Growth Regulators/Growth Hormones

Exogenous application of plant growth regulators/growth hormones (PGRs) has been found to improve plant stress tolerance and increase growth processes (Liu et al., 2019). Growth hormones are identified as playing a critical role in maintaining the plant morphology, flower blooming, development, photosynthetic activity, and stomatal closure in terms of physiological functions in C_4_ plants (Sharma et al., 2020). Exogenous growth hormones were also used to control seed germination, root elongation, cell development, and tiller formation in C_4_ plants cultivated under trace-metal contaminated soils (Maghsoudi et al., 2019). Similarly, in cereals like maize foliar application of plant growth hormones under abiotic stresses considerably improved the leaf area, plant growth, dry biomass, and stem diameter of C_4_ plants (Tran and Popova, 2013; Qandeel et al., 2020). It was reported that exogenous application of various types of brassinosteroids, i.e., 28-homobrassinolide, and 24-epibrassinolide significantly enhanced the biomass and productivity of maize, sugarcane, and sorghum grown under abiotic stresses, i.e., drought, salinity, and trace-metal contaminated soils (Tanveer et al., 2018, 2019). In another study, it was reported that application of 1-amino-cyclopropane-1-carboxylic-acid (ACC-deaminase), humic acid, and oxalic acid considerably enhanced bacterial community development in the rhizosphere, facilitating the remediation of organic pollutants, and improved the plant biomass, which had previously been hindered by the presence of organic contaminants in soil (Ping et al., 2006; Wen-Jie et al., 2011). Similarly, plant growth regulators like melatonin and indole acetic acid are also reported to significantly improve the plant biomass under various abiotic stresses (Rostami et al., 2021). Likewise, the application of abscisic acid, salicylic acid, auxins, cytokinin, methyl jasmonate, and ethylene are also documented to significantly improve the plant biomass under abiotic stresses (Hasan et al., 2019). Yet, to explain precise processes related to the impacts of growth hormones on plant biomass, integrative studies combining conventional and sequencing techniques are required.

## Concluding Remarks and Prospects

Biofuels being an alternative to fossil fuels are considered an integral part of sustainable energy generation systems. To develop the biofuel industry on a sustainable basis, increasing plant biomass is a prime goal as feedstock in the biofuels industry. Biomass accumulation is a complex biological trait. However, advancements in genetics and biotechnology have deciphered that a plethora of genes are controlling growth and development starting from the cell cycle to the juvenile, vegetative, and reproductive maturity phase. Most of the pathways discussed highlight the important genes which have been exploited to tailor the bioenergy crops. Among them, genes involved in the cell cycle, cell wall, and hormone and related transcriptional factors considerably modify the carbohydrate allocation and improve photosynthetic efficiency. But the real challenge is the successful introduction of bioenergy specialized crops in fields on a sustainable basis. Moreover, we pointed out altered regulatory patterns of transcription activity of MYB TFs in C_3_ and C_4_ crops which indicate the lineage-specific carbohydrate storage biopolymer incorporation in both. Therefore, research focuses should be directed on C_4_ crops considering only C_4_ models in terms of biomass accumulation and later on energy generation.

Regarding environmental factors which are acting upon biomass accumulation, CO_2_, light, and temperature are among unavoidable stresses to threaten the growth process. For this, architecture for the maximum light interception, nutrient absorption traits, and certain anatomical changes can be engineered in wild plants to enable the cultivation of bioenergy and orphan lignocellulose crops on marginal lands (resource-deprived). Furthermore, optimization of locality-based bioenergy crops and cultural practices to enhance biomass is critical but not yet elaborated. The above-mentioned tools and practices including breeding, molecular methods (DNA-free genome editing method CRISPR/Cas9), high throughput sequencing, and cultural practices can be opted for engineering and validation of multiple genes from different pathways to generate climate-smart energy crops. The afore-mentioned strategies will only be realistic if they are part of an integrated approach to agriculture that is developed collaboratively with agronomists, engineers, and farmers to contribute to a bio-based economy.

## Author Contributions

NA and RM conceptualized the review. NA wrote the review with the assistance of FH and MF. Habiba and NA designed the figures and tables. YZ improved and revised. All authors finally revised and approved the manuscript.

## Conflict of Interest

The authors declare that the research was conducted in the absence of any commercial or financial relationships that could be construed as a potential conflict of interest.

## Publisher’s Note

All claims expressed in this article are solely those of the authors and do not necessarily represent those of their affiliated organizations, or those of the publisher, the editors and the reviewers. Any product that may be evaluated in this article, or claim that may be made by its manufacturer, is not guaranteed or endorsed by the publisher.

## Abbreviations

ASEAN: Association of South East Asian Nations
eCO_2_: Equivalent CO_2_
GHGs: Greenhouse Gases
mg/h: megagrams per hectare
PEP: Phosphoenolpyruvate
RUBISCO: Ribulose Bisphosphate Carboxylase/Oxygenase
SQUAMOSA: SQUAMOSA Promoter-Binding Protein-Like (SPL)
TFs: Transcription factors.
